# Lime in the Urine

**Published:** 1889-05

**Authors:** Otto Betz

**Affiliations:** Heilbronn, Germany


### LIME IN URINE.

By Dr. Otto Betz, Heilbronn, Germany.

[For Daniel's Texas Medical Journal.]

LAST FALL a fellow-physician complained to me that for sometime past he had noticed a strange deposit in his freshly passed urine. The specimen sent me had been standing for some time and consisted of equal parts of day and night urine; it was of straw-yellow color, with a white sediment about one-fourth of the entire volume.

On boiling the sediment did not disappear. In the clear layer no further turbidity appeared. On adding acids (muriatic, nitric, carbolic acids,) the specimen, while effervescing cleared up completely. Test for albumen and sugar—nil. Under the microscope the sediment appeared a crumby, strongly refractory and amorphous mass, that dissolved, giving off gas bubbles on addition of muriatic acid. It was carbonate of calcium. Inquiry showed that patient had never been sick severely until the time of the Franco-Prussian war ('70–'71), where, after getting over a severe attack of typhus, he contracted a very painful phlebitis of the right leg. Since that time he has been a "morphine eater." Several attempts to stop him from using it, proved unsuccessful. This summer he lost, within a few weeks and without apparent cause, flesh to the amount of twenty pounds or more. At the same time appeared the calcareous deposit in the urine. The sediment was especially heavy after eating of plum or pear sauce. After the use of beer and wine it was much less than usual. The patient had never taken cocaine as a substitute for morphine; but had used some, subcutaneously, to open abscesses on his legs and allowed it to drop into the wound. Neither the amount used, at one time or altogether, in 24 hours, could be ascertained; more than likely it was considerable, as the patient never cut the abscesses except under anæsthesia and the abscesses were numerous. The examination of the thirty-nine year old patient showed: that he was very thin and lean; anemia of the mucous membranes; in the internal organs no anomaly; he never had gonorrhœa, etc.; under the miscroscope the blood showed no change in the relative amount of the red and white blood corpuscles; they soon lost their round form and assumed crenate or zigzag outlines, besides there were many blood discs present.

Concerning the therapeutic treatment, I could not think of attempting to wean the patient of the morphine, as he was physically too reduced and weakened. Thinking of restoring the loss of lime, I ordered phosphate and bicarbonate of sodium, equal parts, as much as could be laid on the point of a knife, three times a day. Levica water for anæmia, good food and regular use of alcoholic drinks. After a few weeks, the carbonate of lime disappeared entirely from the urine. The physical condition of the patient was never excited; the psychic action a little depressed; some loss of memory, an apathetic, dreamy condition existed for a while. This case shows as has also been otherwise observed, that morphine users grow weak and lose flesh rapidly, as soon as they use cocaine.

Lime in the urine was also frequently observed in diseases of the brain and of the spinal chord, mental overwork, nervous prostration, sexual transgressions, (gonorrhœa chronic) hypochondria, chronic diseases of the joints, and lastly, with seemingly healthy individuals, especially in summer time. (S. Laache; Urinalysis 1885—Vogel, Leipsic.) Dr. A. Peyer. "The Microscope at the Sickbed." Basel, 1884, says: An increased discharge of lime is pretty regularly present in cases of diabetes mellitus and with consumption. During fever, there seems to be a decrease of the relative quantity of lime. W. Zuelzer—Textbook on Urinalysis, Berlin, 1880: "During convalescence, after the use of morphine, etc., the quantity of lime is increased."

